# Macroprolactinemia: Diagnostic, Clinical, and Pathogenic Significance

**DOI:** 10.1155/2012/167132

**Published:** 2012-12-04

**Authors:** Akira Shimatsu, Naoki Hattori

**Affiliations:** ^1^Clinical Research Institute, National Hospital Organization Kyoto Medical Center, 1-1 Mukaihata-cho, Fukakusa, Fushimi-ku, Kyoto 612-8555, Japan; ^2^Department of Pharmaceutical Sciences, Ritsumeikan University, Shiga 525-8577, Japan

## Abstract

Macroprolactinemia is characterized by a large molecular mass of PRL (macroprolactin) as the main molecular form of PRL in sera, the frequent elevation of serum PRL (hyperprolactinemia), and the lack of symptoms. Macroprolactin is largely a complex of PRL with immunoglobulin G (IgG), especially anti-PRL autoantibodies. The prevalence of macroprolactinemia is 10–25% in patients with hyperprolactinemia and 3.7% in general population. There is no gender difference and a long-term followup demonstrates that macroprolactinemia develops before middle age and is likely a chronic condition. Polyethylene-glycol- (PEG-) precipitation method is widely used for screening macroprolactinemia, and gel filtration chromatography, protein A/G column, and I125-PRL binding studies are performed to confirm and clarify its nature. The cross-reactivity of macroprolactin varies widely according to the immunoassay systems. The epitope on PRL molecule recognized by the autoantibodies is located close to the binding site for PRL receptors, which may explain that macroprolactin has a lower biological activity. Hyperprolactinemia frequently seen in macroprolactinemic patients is due to the delayed clearance of autoantibody-bound PRL. When rats are immunized with rat pituitary PRL, anti-PRL autoantibodies are produced and hyperprolactinemia develops, mimicking macroprolactinemia in humans. Screening of macroprolactinemia is important for the differential diagnosis of hyperprolactinemia to avoid unnecessary examinations and treatments.

## 1. Introduction

Prolactin (PRL) is an anterior pituitary hormone that plays an important role in lactation during pregnancy but has many other biological functions such as osmoregulation, angiogenesis, and immunoregulation [1]. PRL facilitates the maturation of T cells via IL-2 receptor expression, impairs B cell tolerance to self-antigens through the anti-apoptotic effect, develops antigen-presenting cells, and enhances immunoglobulin production [2]. The increase in serum PRL concentrations (hyperprolactinemia) often develops symptoms such as amenorrhea and galactorrhea in women and impotence in men. It is caused physiologically by pregnancy and pathologically by PRL secreting pituitary adenoma (prolactinoma), hypothalamic and pituitary diseases compressing pituitary stalk, antidopaminergic drugs, hypothyroidism, chest wall diseases, and hepatorenal disorders [3]. However, 29% of hyperprolactinemia has been classified as “idiopathic” because the causes are unknown [4]. Microadenomas in the pituitary gland that cannot be detected by computed tomography (CT) or magnetic resonance imaging (MRI) have been postulated to enter this category.

Anti-PRL autoantibody was found to be one of the major causes of “idiopathic” hyperprolactinemia [5]. It binds to PRL (molecular mass of 23 kDa) forming a large immune complex of PRL with IgG (macroprolactin) and tends to increase serum PRL concentrations. Macroprolactinemia is defined as having macroprolactin (molecular mass greater than 150 kDa) in the predominant molecular form of PRL in sera.

There are reportedly several autoantibodies against hormones other than PRL: insulin [6], glucagon [7], thyroid hormone [8], parathyroid hormone [9], anterior pituitary hormones such as adrenocorticotropic hormone (ACTH) [10], luteinizing hormone (LH), follicle stimulating hormone (FSH) [11], growth hormone (GH) [12] and thyroid stimulating hormone (TSH) [13], and posterior pituitary hormones such as vasopressin and oxytocin [14]. This paper focuses on the diagnostic, clinical, and pathogenic features of macroprolactinemia.

## 2. Diagnosis of Macroprolactinemia

**Figure 1:**
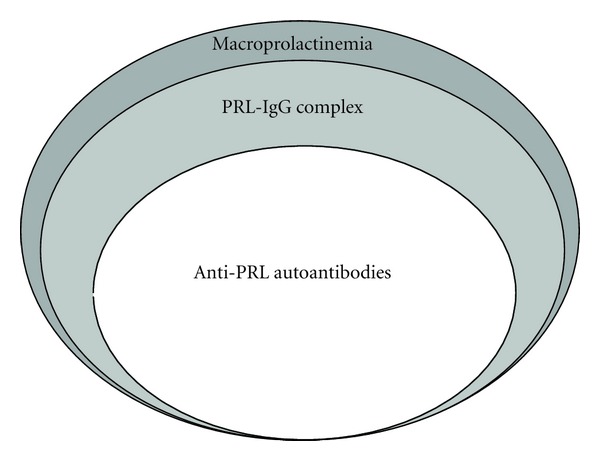
Macroprolactinemia, IgG-bound PRL, and anti-PRL autoantibodies. Macroprolactinemia is a heterogeneous condition with different etiologies; 87% of macroprolactin was PRL-IgG complex and 67% of macroprolactin was autoantibody-bound PRL [15]. Although anti-PRL autoantibody-bound PRL is a major form of PRL-IgG complex and PRL-IgG complex is a major form of macroprolactin, there may be PRL-IgG complex other than autoantibodies and macroprolactin other than PRL-IgG complex as shown in grey area. The diagnosis of macroprolactinemia is made based on the results of PEG-precipitation method or gel filtration chromatography, IgG-bound PRL by protein A or protein G column method, and anti-PRL autoantibody-bound PRL by ^125^I-PRL binding study.

Macroprolactin is mostly a complex of PRL with IgG, especially anti-PRL autoantibodies (Figure 1). The screening of macroprolactinemia is performed by polyethylene-glycol- (PEG-) precipitation method, and the confirmative and qualitative examinations include gel chromatography, protein A/G column, and ^125^I-PRL binding studies [15]. Advantage and disadvantage of these methods are summarized in Table 1.

### 2.1. Polyethylene-Glycol- (PEG-) Precipitation

Since we first applied PEG precipitation method, which had been used to detect anti-insulin autoantibodies, to diagnose macroprolactinemia due to anti-PRL autoantibodies [5], this method has been used for the screening of macroprolactinemia because of its simplicity [19–28]. The method was validated against gel filtration chromatography, a gold standard for the diagnosis of macroprolactinemia [19]. To determine free PRL concentrations, serum samples (50 *μ*L) are mixed vigorously with 50 *μ*L of cold PEG (molecular weight 6000, 25% in water) and centrifuged at 9,100 ×g for 10 min to remove macroprolactin. Serum samples are treated identically, but with water instead of PEG, to determine total PRL concentrations. The PEG-precipitable PRL (%), which represents the amount of macroprolactin, is calculated as follows: (total PRL-free PRL)/total PRL × 100. PEG-precipitation ratio greater than 60% (recovery less than 40%) is used as the cut-off value for the diagnosis of macroprolactinaemia. Gibney et al. [23] proposed an alternative presentation, that is, showing absolute values of free PRL in the supernatant after PEG precipitation. When PRL levels after PEG precipitation fall within a reference range derived from similarly treated normal sera, this is considered a normal result. From a clinical point of view, this is reasonable because such presentation can disclose the patients who need further examinations and treatments for hyperprolactinemia. Both presentations (PEG-precipitation ratio and free PRL value) may be desirable until the time when it is clarified that anti-PRL autoantibodies in macroprolactinemia do not affect PRL actions and macroprolactinemia is totally a benign condition.

As to the prevalence of macroprolactinemia screened by PEG-precipitation method, it is noteworthy that the detectability of macroprolactin varies a lot according to the PRL assay systems [28]. It is possible that some reagent antibodies against PRL in assay kits can recognize anti-PRL autoantibody-bound PRL and others not. Moreover, it may be attributable to the heterogeneity of macroprolactinemia [17, 29].

### 2.2. Gel Filtration Chromatography

**Figure 2:**
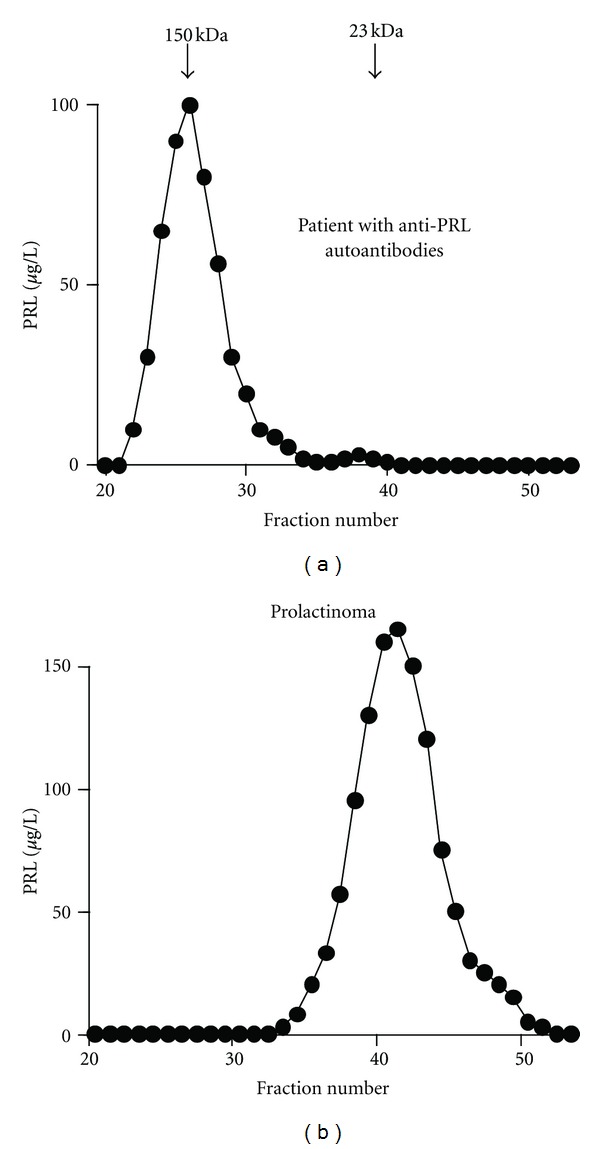
Gel filtration chromatography of macroprolactin and prolactin. Representative gel filtration chromatography of PRL in serum samples of macroprolactinemia (a) and prolactinoma (b). (Reproduced from [16]).

Traditionally, gel filtration chromatography has been used to separate various molecular forms of PRL: little (monomeric) PRL (molecular size: 23 kDa), big PRL (45–50 kDa), and big-big PRL (more than 100 kDa) [30]. Little PRL is a major form of pituitary and serum PRL, big PRL is a dimer of little (monomeric) PRL, and big-big PRL has been believed to be an aggregate of monomeric PRL. Macroprolactin is defined as a large molecular-sized PRL greater than 100 kDa that is included in big-big PRL, and the state in which the ratio of macroprolactin is substantially increased in sera is called macroprolactinemia (Figure 2). There is no a clear-cut value of “substantial increase”, but conventionally a diagnosis of macroprolactinemia is made when more than 30–60% of PRL is in the macroprolactin form of gel filtration chromatography [27]. Although gel filtration chromatography was regarded as the gold standard for the diagnosis of macroprolactinemia, it is time, labor, and cost consuming. Thus this method is used to confirm the diagnosis of macroprolactinemia.

### 2.3. Protein A/G Column

Protein A binds to the Fc portion of immunoglobulin molecules without interfering with the antigen-binding site. Protein G binds only to IgG and its subclasses, separating out IgA, IgM, IgD, and albumin, which may bind to protein A [31]. These reagents are used to identify macroprolactin due to PRL-IgG complexes, and most patients with macroprolactinemia belong to this category [15, 17].

### 2.4. ^125^I-PRL Binding Study

Serum samples (100 *μ*L) and ^125^I-PRL (20000 cpm/50 *μ*L sodium phosphate buffer containing 0.1% bovine serum albumin and 0.1 mol/L NaCl) were incubated for 1 h at room temperature. After incubation, 200 *μ*L of cold 25% (wt/wt) PEG 6000 (final concentration 12.5%) was added, and the mixture was mixed vigorously and centrifuged at 3000 rpm for 20 min. The sediment was washed once with 12.5% PEG, and the radioactivity was measured with *γ*-counter [5, 15, 17].

## 3. Clinical Significance

### 3.1. Prevalence of Macroprolactinemia

**Figure 3:**
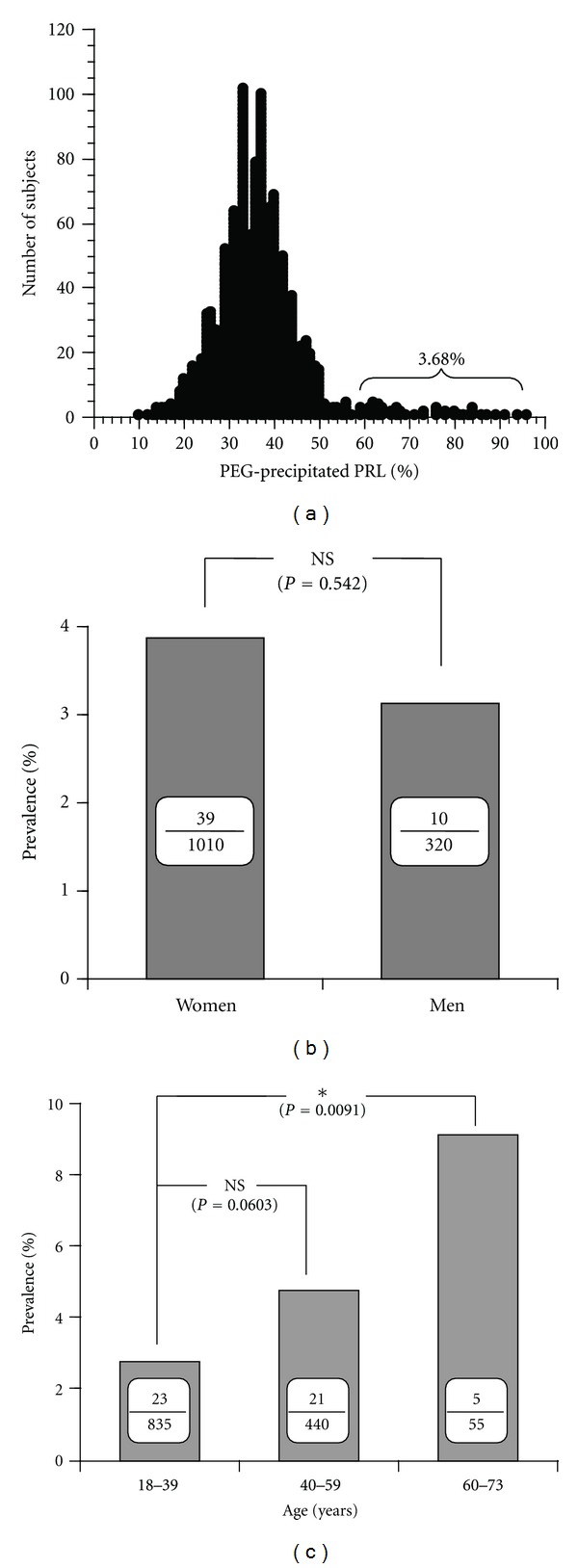
The prevalence of macroprolactinemia and its gender and age dependency in normal subjects. (a) The circle represents the value of PEG-precipitation ratio in each individual and the vertical line shows the number of subjects. The graph shows almost a normal distribution except for a subgroup (macroprolactinemia) having the ratio greater than 60% (mean + 2SD). (b) The prevalence of macroprolactinemia in women and men. (c) The prevalence of macroprolactinemia in different age groups. (Reproduced from [17]).

Figure 3 shows the prevalence of macroprolactinemia and its gender and age dependency in a large group of hospital workers (*n* = 1330) [17]. Macroprolactinemia is present in 3.68% in general population. The prevalence is not different between women and men, and it tends to increase in elderly people. In patients with hyperprolactinemia, the prevalence of macroprolactinemia is reportedly 10–25% [20–23].

### 3.2. Symptoms

Hyperprolactinemia tends to develop in patients with macroprolactinemia [17] because of the delayed clearance of macroprolactin. Although hyperprolactinemia frequently causes menstrual irregularities and galactorrhea in women and loss of libido in men, patients with macroprolactinemia often lack such clinical symptoms of hyperprolactinemia [18]. *In vitro* experiment using T47D human breast cancer cells, the bioactivity of autoantibody-bound PRL was found to be low [32], compatible with the clinical characteristics. However, controversial results are also reported [22] probably because the prevalence of macroprolactinemia is so high that the patients with macroprolactinemia might have other causes of hyperprolactinemia as well [27]. Women with macroprolactinemia can get pregnant and deliver normal babies without any treatments of hyperprolactinemia [33]. Long-term follow-up studies of macroprolactinemia show that macroprolactinemia persisted but no symptomatic progression was noted [15, 34]. Thus it is strongly recommended that all patients with hyperprolactinemia should take the screening test of macroprolactinemia in order to avoid unnecessary examinations and treatments.

### 3.3. Association with Other Autoimmune Disorders

Anti-PRL autoantibodies are a major cause of macroprolactinemia. Thus it is possible that some autoimmune disorders might be accompanied with macroprolactinemia. There were some case reports showing the association between macroprolactinemia and autoimmune thyroid disorders such as Graves' disease and Hashimoto's thyroiditis [35, 36]. Hyperprolactinemic SLE patients reportedly had a higher frequency of macroprolactinemia (14/43, 32.6%) [37]. However, other studies examining a large number of patients revealed no specific association between macroprolactinemia and autoimmune disorders [22, 38]. It is likely that autoimmune mechanisms may be directed mainly toward prolactin molecule in macroprolactinemia.

## 4. Pathogenic Significance

### 4.1. Characteristics of Anti-PRL Autoantibodies

**Figure 4:**
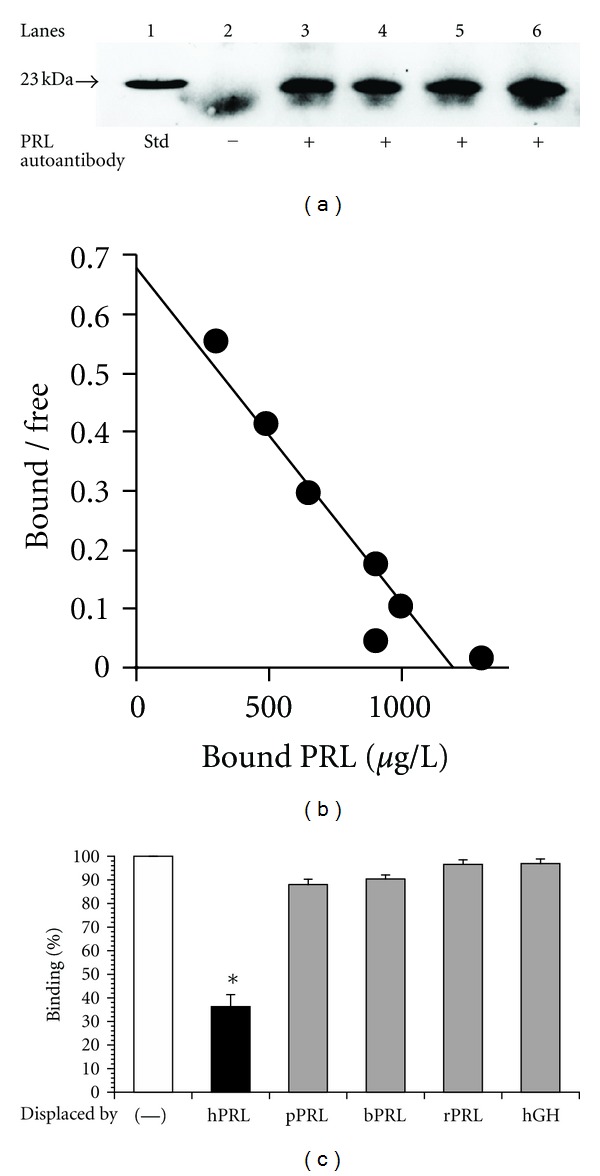
Electrophoresis of macroprolactin and Scatchard analysis of anti-PRL autoantibodies. (a) IgG was purified from the sera of the patients with macroprolactinemia (lanes 3–6) using a protein G column and run on SDS-PAGE under a nonreducing condition. PRL, which bound to the autoantibodies, was dissociated and immunostained at the same position as the 23 kDa human PRL standard (lane 1). The 23 kDa PRL band was not observed when IgG from a patient with prolactinoma was used (lane 2). The fuzzy bands migrating faster than PRL may be nonspecific staining of IgG light chain. (b) Scatchard analysis revealed low-affinity and high-capacity autoantibodies. (c) Displacement of ^125^I-hPRL-autoantibody complex by human PRL and other related peptides; hPRL: human PRL, pPRL: porcine PRL, bPRL: bovine PRL, rPRL:r at PRL, and hGH: human GH. Only hPRL could displace the binding of ^125^I-PRL with the autoantibodies. (Reproduced from [5, 15, 18]).

When IgG was purified from sera using protein G column, a significant amount of PRL was copurified with IgG in patients with macroprolactinemia. When this IgG fraction was analyzed on SDS-PAGE under nonreducing condition, the IgG-bound PRL was dissociated and immunostained at the same position as the 23 kDa human PRL standard as shown in Figure 4(a), suggesting that PRL is noncovalently bound to IgG [18]. Scatchard analysis revealed low-affinity and high-capacity autoantibodies as shown in Figure 4(b) [5, 33] and only human PRL could displace the binding of ^125^I-PRL and anti-PRL autoantibodies as shown in Figure 4(c) [15, 39], suggestive of the specificity of the autoantibody to human PRL. The subclass of anti-PRL autoantibodies was mainly IgG4, suggesting that chronic antigen stimulation may be involved [40].

### 4.2. Mechanisms of Hyperprolactinemia

Dopamine or bromocriptine, dopamine D_2_ receptor agonist, decreases serum PRL concentrations [1]. Administration of these drugs to the patients with macroprolactinemia did not decrease serum PRL concentrations so much as in prolactinoma, suggesting that the clearance of PRL is delayed [33]. This was confirmed by the animal experiments showing that anti-PRL autoantibody-bound PRL was cleared from the rat circulation more slowly than monomeric PRL [18] and that hyperprolactinemia developed in animal model of macroprolactinemia [41]. Moreover, a significant positive correlation was present between anti-PRL autoantibody titers and serum PRL concentrations in humans [33], suggesting that anti-PRL autoantibody is a cause of hyperprolactinemia. It is assumed that the hypothalamic negative feedback mechanism by autoantibody-bound PRL does not work because the complex cannot access to the hypothalamus and/or anti-PRL autoantibodies interfere with the binding of PRL to the receptor. Thus, serum PRL concentrations may increase until free PRL concentrations exceed normal PRL concentrations, when negative feedback mechanisms begin to operate to make free PRL levels down. Actually, free PRL concentrations in sera from most patients with macroprolactinemia are within normal range [17, 27].

### 4.3. Mechanisms of Low Bioactivity

Macroprolactinemia is characterized by a lack of clinical symptoms of hyperprolactinemia and several studies using different PRL bioassay systems showed that the bioactivity of macroprolactin is low [32, 42, 43]. The epitope mapping using deleted PRL fragments revealed that the binding sites of PRL molecule to anti-PRL autoantibodies reside in N and C terminals of PRL [39]. The core human PRL structure is made up of four major *α*-helices. The *α*-helix groups are in two antiparallel pairs, helix 1/helix 4 and helix 2/helix 3, each pair being packed more closely together. Therefore, several N- and C-terminal amino acid residues are located closely in the three-dimensional structure forming a part of binding site 1 to human PRL receptors [44]. Thus, anti-PRL autoantibodies and PRL receptors bind to the similar regions on PRL molecule, raising a possibility that the autoantibodies may compete the binding of PRL molecule to its receptors, resulting in the low biological activity.

### 4.4. Possible Causes of Anti-PRL Autoantibody Production

Mechanisms involved in the development of anti-PRL autoantibodies are unknown. Genetic susceptibility and environmental factors may alter the immune response in hosts as postulated in other autoimmune disorders [45]. On the other hand, some changes in PRL molecule may increase the antigenicity leading to the production of anti-PRL autoantibodies. The finding that IgG4 was a predominant subtype of anti-PRL autoantibodies supports the latter possibility because IgG4 response occurs in chronic antigen response. Switch from IgG1 to IgG4 response is driven by the repeated exposure to allergens, and IgG4 is a predominant subclass in other autoantibodies [46, 47]. Posttranslational modifications such as phosphorylation of PRL might be involved in the possible altered antigenicity [40].

## 5. Conclusion

Macroprolactinemia should be examined in all serum samples with hyperprolactinemia for the differential diagnosis because it is one of the major causes of hyperprolactinemia. Neither medications nor further examinations are recommended if free PRL concentrations after precipitating macroprolactin with PEG are normal, because the biological activity of macroprolactin is low and pregnancy is possible without any treatment for hyperprolactinemia. Macroprolactinemia is a heterogeneous condition with different etiologies and further study is necessary to glean it in its entirety.

## Figures and Tables

**Table 1:** Advantage and disadvantage of methods for the diagnosis of macroprolactinemia.

	Advantage	Disadvantage
Polyethylene glycol (PEG)	Simple and inexpensive	Not highly specific
	Screening for macroprolactinemia	

Gel chromatography	Accurate Confirming macroprolactinemia	Time consuming and expensive

Protein A/G	Identifying IgG-bound PRL (common cause of macroprolactin)	Expensive

^ 125^I-PRL binding	Identifying anti-PRL autoantibodies (common cause of IgG-bound PRL)	Time consuming and hazardous Needs radio isotope facilities
